# Anhedonia and emotional numbing in treatment-seeking veterans: behavioural and electrophysiological responses to reward

**DOI:** 10.1080/20008198.2018.1446616

**Published:** 2018-03-15

**Authors:** Kasper Eskelund, Karen-Inge Karstoft, Soren B. Andersen

**Affiliations:** a Department of Military Psychology, Danish Veteran Centre, Danish Defence, Copenhagen, Denmark; b Research and Knowledge Centre, Danish Veteran Centre, Danish Defence, Ringsted, Denmark; c Department of Psychology, University of Copenhagen, Copenhagen, Denmark

**Keywords:** Veterans, anhedonia, emotional numbing, posttraumatic stress, EEG, ERP, veteranos, anhedonia, entumecimiento emocional, estrés postraumático, EEG. ERP, 老兵, 快感缺失, 情绪麻木, 创伤后应激, EEG, ERP, • Anhedonia and emotional numbing are frequently seen in the wake of trauma.• In depression, anhedonia has been linked to reward processing, but this mechanism is not well understood in PTSD.• In treatment-seeking veterans, we find that anhedonia is linked to reward anticipation while emotional numbing is linked to reward consummation, mirrored in frontal EEG-activity.• Disentangling anhedonia and emotional numbing can improve diagnosis and inform treatment of veterans.

## Abstract

**Background**: Anhedonia is a common symptom following exposure to traumatic stress and a feature of the PTSD diagnosis. In depression research, anhedonia has been linked to deficits in reward functioning, reflected in behavioural and neural responses. Such deficits following exposure to trauma, however, are not well understood.

**Objective**: The current study aims to estimate the associations between anhedonia, PTSD symptom-clusters and behavioural and electrophysiological responses to reward.

**Methods**: Participants (*N* = 61) were recruited among Danish treatment-seeking veterans at the Department of Military Psychology in the Danish Defence. Before entering treatment, participants were screened with symptom measurement instruments and participated in a joint behavioural-electrophysiological experiment. The experimental paradigm consisted of a signal-detection task aimed at assessing reward-driven learning. Simultaneous electrophysiological-recordings were analysed to evaluate neural responses upon receiving reward, as indicated by the Feedback-Related Negativity (FRN) component.

**Result**: Anhedonia as conceptualized in depression correlated with behavioural learning (*r* = -0.28, *p* = .032). Neither anhedonia nor behavioural learning correlated with FRN. However, the anhedonia symptom cluster of PTSD did correlate with FRN (*r* = 0.29, *p* = .023). Extending upon this in an exploratory analysis, the specific PTSD-symptom emotional numbing was found to correlate moderately with FRN (*r* = 0.38, *p* = .003).

**Conclusion**: The present data suggest that anhedonia in trauma-exposed individuals is related to the anticipatory aspect of reward, whereas the neural consummatory reward response seems unlinked. Interestingly, emotional numbing in the same population is related to the consummatory phase of reward, correlating with the FRN response. This suggests that anhedonia and emotional numbing in response to trauma might pertain to different phases of reward processing.

## Abstract (es)

**Planteamiento:** La anhedonia es un síntoma frecuente después de la exposición al estrés traumático y una característica del diagnóstico de TEPT. En la investigación de la depresión, la anhedonia se ha relacionado con los déficits en el funcionamiento de la recompensa, que se refleja en las respuestas conductuales y neuronales. Dichos déficits después de la exposición al trauma, sin embargo, no se entienden bien.

**Objetivo:** El presente estudio tiene como objetivo estimar las asociaciones entre la anhedonia, los grupos de síntomas de TEPT y las respuestas conductuales y electrofisiológicas a la recompensa. Métodos: Los participantes (*N* = 61) fueron reclutados entre los veteranos daneses que buscaban tratamiento en el Departamento de Psicología Militar de la Defensa Danesa. Antes de empezar al tratamiento, los participantes fueron evaluados con instrumentos de medición de síntomas y participaron en un experimento conjunto electrofisiológico-conductual. El paradigma experimental consistió en una tarea de detección de señales destinada a evaluar el aprendizaje basado en recompensas. Simultáneamente se analizaron las grabaciones de EEG para evaluar las respuestas neurales al recibir la recompensa, según lo indicado por el componente de negatividad relacionada con la retroalimentación (FRN, por sus siglas en inglés).

**Resultados:** La anhedonia, tal como se conceptualizó en la depresión, se correlacionó con el aprendizaje conductual (*r* = −0.28, *p* = .032). Ni la anhedonia ni el aprendizaje conductual se correlacionaron con la FRN. Sin embargo, el grupo de síntomas de anhedonia del TEPT se correlacionó con el FRN (*r* = 0.29, *p* = .023). Sobre la base de esto en un análisis exploratorio, el entumecimiento emocional específico de los síntomas del TEPT se correlacionó moderadamente con la FRN (*r* = 0.38, *p* = .003).

**Conclusiones:** Los datos actuales sugieren que la anhedonia en individuos expuestos al trauma está relacionada con el aspecto anticipatorio de la recompensa, mientras que la respuesta de recompensa neural consumada parece desvinculada. Curiosamente, el adormecimiento emocional en la misma población está relacionado con la fase de recompensa consumada, que se correlaciona con la respuesta FRN. Esto sugiere que la anhedonia y el entumecimiento emocional en respuesta al trauma podrían pertenecer a diferentes fases del procesamiento de la recompensa.

## Abstract (zh)

**背景：**快感缺失是创伤应激暴露后的一个普遍症状，也是 PTSD 的一个诊断特征。在抑郁研究中，快感缺失和行为和神经反应体现的奖赏功能缺失联系在一起。而创伤暴露后的这种缺失还没有被很好理解。

**目标：**本研究旨在考查快感缺失、PTSD 症状簇和对奖赏的行为以及电生理反应之间的关联性。

**方法：**参与者（*N* = 61） 是到丹麦国防部的军人心理科寻求治疗的老兵。在治疗前，使用症状测量工具对被试进行筛查，被试还参加了一个行为-电生理实验。该实验范式包括一个用于评估奖赏驱动学习的信号检测任务。任务同时用 EEG 记录用来分析接受奖赏时候的神经反应（使用反馈相关负成分FRN）。

**结果：**抑郁中的快感缺失和行为学习有关(*r* = -0.28, *p* = .032)。快感缺失和行为学习都与 FRN不相关。但是， PTSD 的快感缺失症状簇和 FRN 相关(*r* = 0.29, *p* = .023)。 在此基础上进行一个扩展分析，发现PTSD 特异的症状——情绪麻木也和 FRN 呈现中等相关(*r* = 0.38, *p* = .003)。

**结论：**本研究数据说明快感缺失在创伤暴露个体中是和奖赏的预期有关，但和奖赏的实际神经反应似乎无关。有意思的是，同样人群的情绪麻木和奖赏的实际神经反应（FRN反应）有关。这说明创伤反应中的快感缺失和情绪麻木可能属于不同的奖赏加工过程。

## 1. Introduction

Veterans seeking treatment for psychological problems elicited by deployment to war zones present with a variety of post-traumatic stress (PTS) symptoms. Among these are negative alterations of mood and cognition, a core symptom cluster of posttraumatic stress disorder (PTSD) in the DSM-5 (APA, 2013). Symptoms in this cluster such as anhedonia and emotional numbing have previously been shown to distinguish between PTSD patients with moderate and severe symptom levels (Breslau, Reboussin, Anthony, & Storr, 2005). The presence of such symptoms has further been suggested as a severity marker of the disorder (Naifeh, Richardson, Del Ben, & Elhai, 2010).

In recent factor analytic studies of PTSD, anhedonia has been found to constitute an independent symptom cluster of PTSD (Armour et al., 2015; Pietrzak et al., 2015). Specifically, in a 7-factor model of PTSD, anhedonia consists of three symptoms, namely loss of interest, detachment and restricted range of affect (Armour et al., 2015). This anhedonia symptom cluster has been shown to be strongly associated with current depression, reduced mental functioning and quality of life as well as increased suicidal ideation (Pietrzak et al., 2015). As such, anhedonia might not only suggest severe PTSD, but constitute a comorbidity link between PTSD and depression and be a useful predictor of mental dysfunction and reduced quality of life in trauma survivors.

In depression research, anhedonic depression has been suggested as a specific phenotype (Pizzagalli, Jahn, & O’Shea, 2005). Further, in the Research Domain Criteria (RDoC) framework, anhedonia has been suggested as a core construct in the cross-diagnostic study of psychopathology (Cuthbert, 2014). Hence, anhedonia might constitute a crossover phenotype between the affective and the trauma-related spectrum. The abovementioned findings motivate further research into symptoms bridging posttraumatic stress and depression such as anhedonia (Afzali et al., 2017).

Anhedonia has been linked to reward functioning and deficits therein (Der-Avakian & Markou, 2012). Reward functioning is the ability to feel pleasure when consuming or collecting stimuli of positive valence. Further, it implies being motivated for obtaining such stimuli and promoting behaviour that produces them. As such, reward is pivotal for driving many human behaviours. Following this, different phases of reward functioning may be defined as distinct behaviours (Berridge & Kringelbach, 2008; Nawijn et al., 2015). *Reward wanting* is the anticipatory motivation towards obtaining the stimulus, whereas *reward consumption* is linked to the pleasure felt by obtaining the stimulus. *Reward learning* is the ability to increase the amount of obtained stimuli by predicting and performing reward-producing behaviours.

Being involved in fundamental psychological processes such as learning and the ability to seek and experience pleasure, impaired reward processing may alter behaviour and well-being of affected individuals considerably. If gratification by previously enjoyed activities is diminished, or if the desire to optimize behaviours towards collecting more rewards is impeded, it may hamper the fundamental meaning of many goals in everyday or long-term endeavours, such as in the workplace or career, in maintaining or developing relations to others, or in achieving personally meaningful or gratifying goals.

Although reward processing has a clear subjective, experiential aspect, it can be assessed using objective psychophysical and physiological methods. Studying reward functioning and anhedonia in MDD patients as well as healthy controls, Pizzagalli and colleagues (2005) developed a psychophysiological experimental paradigm for measuring reward learning. In a basic signal detection task discriminating between two simple visual stimuli (horizontal line lengths), participants are given feedback and monetary reward on their performance at asymmetric rates over the two stimuli. As reward is suggested to facilitate learning, a higher reward rate would produce a higher outcome in participants with unimpeded reward functioning, whereas a lower reward rate would not support learning and thus produce a lower outcome. Following this, normal reward functioning would show a response bias in performance over the two stimuli, because reward-based learning would enhance detection of the often-rewarded stimulus more. Conversely, in subjects with impeded reward functioning, reward would to a lesser degree support learning, producing a more symmetric detection rate across the asymmetrically rewarded stimuli. Pizzagalli et al. (2005) found the expected negative correlation between response bias towards the rewarded stimulus and level of self-reported anhedonic symptoms in a healthy population. In a subsequent study using the same paradigm to study learning in major depressive disorder (MDD) patients and healthy controls, Pizzagalli and colleagues (2008) found that anhedonic symptoms correlated with impairment in the development of response bias towards the frequently rewarded stimulus.

Santesso and colleagues (2008) investigated neural correlates of reward functioning tied to responses to reward stimuli. Employing a paradigm similar to the task constructed by Pizzagalli and colleagues (2008), their study suggested a moderate correlation between reward-based learning (as expressed in response bias due to reinforcement asymmetry) and a well-known event-related potential (ERP) response to obtained rewards (i.e. reward consumption), the Feedback-Related Negativity (FRN) component (Gehring, 2002). FRN is a negative deflection of the ERP in response to feedback to performance on a given task, and it is hypothesized to reflect evaluation of feedback in a given comparative context (Foti & Hajcak, 2009). This has been understood as mirroring a basic reinforcement prediction and learning mechanism, highly relevant to reward processing (Schultz, 2002). The FRN response is known to vary with the valence of feedback, to the effect that worse-than-expected feedback yields a more negative response. While the FRN is represented in EEG as a negative peak at ~250 ms latency at frontal electrode sites, source-localization studies have related the response to the anterior cingulate cortex (Yeung, Holroyd, & Cohen, 2005).

Comparing depression self-rating scores and FRN in a non-clinical sample, Foti and Hajcak (2009) found a weak correlation between depression symptom level and FRN. Further, their findings suggested similar correlations between FRN and stress but not anxiety scores.

A recent review has described existing research efforts investigating reward functioning in PTSD (Nawijn et al., 2015). While results were mixed, the authors concluded that, in general, decreased reward anticipation and reward consumption responses were observed more often in PTSD-patients compared to controls. The authors highlight the heterogeneity of PTSD (Galatzer-Levy & Bryant, 2013) as a potential explanation for the mixed findings, and suggest that future research should move away from the simple comparison of PTSD-patients versus healthy controls, and instead focus on specific symptoms and symptom clusters and their relation to reward functioning (Nawijn et al., 2015).

### 1.1. Current study

The abovementioned experimental studies suggest a connection between anhedonia and reward learning as a measure of reward functioning. Further, they indicate FRN as a possible neural correlate or marker of reward functioning. In the present study, we employ a paradigm similar to experiments by Santesso et al. (2008) and Pizzagalli et al. (2008), comparing reward-learning through signal-detection performance and neural reward response through FRN.

As noted by Nawijn and colleagues (2015), previous attempts at finding correlates between reward function and symptoms of PTS may have partly been occluded by case-control designs that do not reflect the symptom heterogeneity within the PTS spectrum. In the current study, we take a different approach to the investigation of reward functioning related to PTS. Inspired by the RDoC framework (Cuthbert, 2014), all patients eligible for assessment at a clinic for the treatment of deployment-related problems in Danish veterans are invited to participate in the study during the data collection period of the experiment. The sample is thus a clinical sample of treatment-seeking veterans, but their inclusion is not based on specific symptom patterns or diagnoses. This approach enables investigation of anhedonia as a cross-diagnostic phenotype or symptom dimension, presenting in the variety of psychological difficulties that may develop after deployment to war zones.

Hence, we investigate correlations of response bias in reward learning and reward-elicited FRN with anhedonia in a sample of treatment-seeking veterans from the following hypotheses:

- Level of anhedonia in PTS-affected veterans will correlate negatively with reward learning.
- PTS-affected veterans who fail to learn from reward will produce a shallower FRN compared to veterans who learn from reward.
- Level of anhedonia in PTS-affected veterans will correlate negatively with FRN.

## 2. Material and methods

### 2.1. Recruitment procedure and participants

The study is part of an ongoing data collection at the Department of Military Psychology (DMP) in the Danish Defence. More specifically, all individuals who are considered for treatment at the DMP are thoroughly assessed at treatment baseline with a range of self-report measures of mental health. From this pool of treatment-seeking individuals, participants for the current study were randomly selected. The study draws on data from the baseline assessment and from the testing session consisting of a behavioural paradigm and concurrent EEG recordings. Hence, the sample consists of individuals who provided data from all three data sources (baseline assessment, behavioural paradigm and EEG).

In total, 78 individuals who were invited to participate in the study had completed the baseline assessment and were therefore eligible for inclusion. Of these, 12 did not complete all three rounds of the behavioural paradigm. Further, for five individuals, EEG-data from more than 25% of trials in the ERP paradigm were rejected due to artefacts, and all data from these individuals were therefore excluded. Hence, the final sample consisted of 61 individuals.

The participants had a mean age of 33.5 (*SD* = 7.9) and were mainly male (93.4%). Within the group of participants, 63.3% fulfilled the criteria for probable PTSD (as defined by a score on the PTSD Check-List, Civilian Version (PCL-C; Weathers, Litz, Herman, Huska, & Keane, 1993) ≥ 44; Karstoft, Andersen, Bertelsen, & Madsen, 2014), and 72.7% fulfilled the criteria for severe depression (as defined by a score of ≥ 30 on the Major Depression Inventory; Olsen, Jensen, Noerholm, Martiny, & Bech, 2003).

### 2.2. Stimuli and procedure

At intake, participants filled out a range of symptom rating questionnaires of which the ones that are relevant for the current study are described here. Within two weeks of referral and prior to inclusion in any treatment programme or other interventions, participants underwent a combined psychophysical and EEG paradigm, designed to assess alterations in behavioural and neural responses associated with anhedonia.

### 2.3. Self-reported symptom measures

To assess symptoms of PTSD, we applied the PCL-C. The PCL-C is a 17-item questionnaire where the symptoms of PTSD in DSM-IV are rated on a Likert scale from 1–5 according to how much the participant experienced the symptom during the last month. To arrive at PTSD symptom severity, items are summed to arrive at a total score ranging from 17–85. The PCL-C has been found to have high reliability (α = 0.94; Weathers et al., 1993), which was also the case in our study (α = 0.93). Anhedonia was indicated in PCL with the factor *Anhedonia* (PTSD-anhedonia) as defined by Pietrzak and colleagues (2015) and consisted of the symptoms *loss of interest, detachment* and *restricted range of affect*. Reliability of these three items combined as an anhedonia score was acceptable (α = 0.74).

*Mood and Anxiety Symptoms Questionnaire* (MASQ), *subscale for Anhedonic Depression* (MASQ-AD; Clark & Watson, 1991) was used to assess the broader concept of anhedonia. MASQ-AD is a 22-item questionnaire on symptoms of anhedonia in depression. MASQ-AD has two subscales, loss of interest (eight items, score range 8–40) and positive affect (14 items, score range 14–70) referring to the negative and the positive extremes of the anhedonic spectrum, respectively. Items of the respective subscale are summed and, ultimately, all items are summed to arrive at a total anhedonic depression score (range 22–110). Reliability of the positive affect scale (α = 0.93) and the total anhedonic depression scale (α = 0.91) was excellent, while it was acceptable for the loss of interest scale (α = 0.71). MASQ-AD was included in the current study as to enable a comparison with findings by Pizzagalli and colleagues (2005).

### 2.4. Signal-detection task design

Stimuli were presented on a HP Compaq L1950 display and an ATI Radeon HD6350 graphics processing unit with a screen refreshment rate of 60 Hz. The stimulus sequence was generated in MATLAB using functions from the Psychophysics Toolbox (Brainard, 1997; Kleiner et al., 2007; Pelli, 1997). Onsets of target stimuli were recorded with a photo diode mounted on the stimulus display. Visual stimulus material was the same as that used by Santesso et al. (2008).

**Figure 1.**
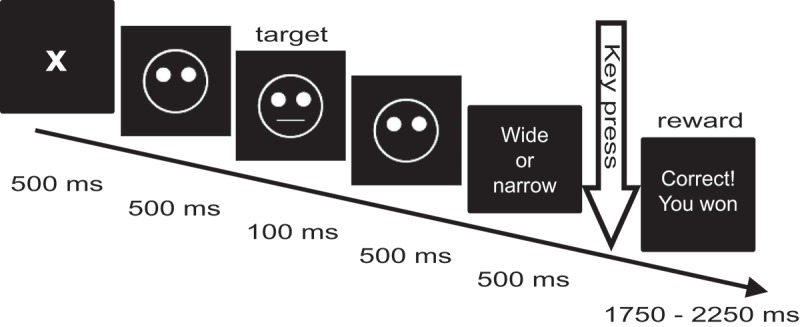
Schematic representation of a single trial in the probabilistic reward learning task in the current study, adapted from Pizzagalli et al. (2005) and Santesso et al. (2008).

The experimental procedure replicated the probabilistic reward signal-detection task previously designed by Tripp and Alsop (1999) and adapted by Pizzagalli et al. (2005, 2008) and Santesso et al. (2008) (see Figure 1). In brief, this task presents 300 trials in three blocks. Each trial presents a fixation cross for 500 ms followed by a mouthless cartoon face (a ‘smiley’) for 500 ms. For 100 ms, a ‘narrow mouth’ or ‘wide mouth’ (short or long horizontal line) is added upon the cartoon face. The distribution of these two lengths is even across trials and the sequence of their appearance is randomized across the stimulus sequence. Upon this, the face is shown mouthless for another 500 ms. Subsequently the participant is asked to indicate if the mouth was wide or narrow by pressing either of two keys (key assignment [left/right] was counterbalanced across subjects) within an interval of 1000 ms. If either key is pressed, the sequence proceeds to either a reward screen displaying the text ‘Correct! You won 0.10 DKK’ or to a blank screen at the next screen refreshment. If the participant does not respond within 1000 ms, the sequence proceeds to a blank screen. The blank screen or reward message is displayed for an interval of randomized duration (range 1750–2250 ms) to reduce entrainment effects on EEG oscillations. Participants were instructed that the total reward sum would be paid in cash after conclusion of the experiment. The mean summed and paid reward per participant was 11.20 DKK, with the maximal possible reward sum being 12.00 DKK (equivalent to ~2 USD).

The reward message is shown as feedback on correctly indicated trials. However, only 40 of 100 trials in a block will produce this reward. Furthermore, these 40 reward trials are asymmetrically distributed with a 10/30 ratio across the two targets (‘narrow mouth’ and ‘wide mouth’), one stimulus providing *rich* feedback, and the other *lean* feedback. The assignment of rich and lean feedback to the two target stimuli is randomized across subjects. In trials with no feedback assigned, or in the case of incorrect responses, a blank screen is displayed.

The psychological mechanism investigated is the response to reward and how reward reinforces learning. However, participants may experience difficulties in adapting to the task, which is not relevant to the reward response in itself. As to expose all participants to a roughly similar number of rewards, the assignment of rewards to target stimuli is adapted during the experiment. Thus, if a participant responds incorrectly in a trial that answered correctly would have produced a reward, the following non-rewarding trial with the same target stimulus is changed to a potentially rewarding trial.

Individual behavioural responses in the signal-detection task were subjected to an analysis revealing response bias towards the *rich* stimulus. Following Pizzagalli et al. (2005), response bias *b* for each block was computed as:

$$logb=\;\frac{1}{2}log\left(\frac{rich\;correct*lean\;incorrect}{rich\;incorrect*lean\;correct}\right).$$

Reward learning was further derived as the difference in response bias developed between Block 1 and Block 3. As Pizzagalli and colleagues (2008) found that the response bias development completed between first and third block was uninformative in their experiment, they further computed reward learning expressed within the first round only. This parameter was derived in the same manner as above, but comparing response bias in the first half of first block with response bias in the latter half of the same block. In the current study, we thus include both of these reward learning measures.

Based on their response bias, participants were allocated to groups of reward-learners and reward-non-learners. Participants developing a response bias of 0 or below were assigned to the reward-non-learners group, whereas participants producing a positive response bias were assigned to the reward-learners group.

### 2.5. EEG recordings and preprocessing

Continuous EEG was recorded from the scalp at 19 sites using an ECI cap with tin electrodes and a MITSAR EEG-201 amplifier system. EEG was sampled at 500 Hz and 16 bit depth with 0.1–150 Hz bandpass filtering during recording and reference to linked ears. Impedance across all electrodes was below 10 kΩ.

Upon recording, continuous EEG was processed within the EEGLAB toolbox for MATLAB (Delorme & Makeig, 2004). EEG data was bandpass filtered (1–30 Hz) offline and the sampling rate was reduced to 100 Hz. Data was epoched relative to onset of the reward stimulus, including data in the interval from 100 ms before stimulus onset to 600 ms after, and for each channel a baseline representing the mean potential of the prestimulus interval was removed from each epoch. All epochs exceeding ± 100 uV at occipital, parietal or central sites were excluded. Subsequently, data was subjected to an ICA algorithm (runica). Independent components representing eye movement artefacts were identified using the EyeCatch toolbox (Bigdely-Shamlo, Kreutz-Delgado, Kothe, & Makeig, 2013). Selected components were removed from the data and the EEG signal reconstructed.

If more than 25% of a participant’s epochs were rejected during preprocessing steps, data from this participant was excluded from the analysis. On behalf of this, data from five subjects were excluded.

ERPs for each subject were computed as the average potential of all epochs remaining after the above preprocessing steps. The FRN was extracted as the most negative peak within the 200–400 ms interval post stimulus at site Fz (Gehring, 2002; Santesso et al., 2008, 2011).

### 2.6. Statistical analyses

We present correlations between our self-report measures (PTSD-anhedonia, MASQ-AD total and subscales) and response bias. Further, we conduct independent *t*-tests to test the FRN-difference between learners and non-learners. Finally, we present correlations between the self-report measures of anhedonia and FRN. ERP-plots are produced for learners vs. non-learners.

## 3. Results

### 3.1. Is the level of anhedonia in PTS-affected veterans negatively correlated with reward learning?

Correlations between self-reported symptoms of anhedonia can be seen in Table 1. Strong intercorrelations between the overall MASQ scale and MASQ subscales were found. PTSD-anhedonia was moderately to strongly correlated with all MASQ-scales. Reward learning based on the response bias developed from Block 1 to Block 3 was not significantly correlated to any of the self-reported anhedonia measures. However, when calculated across Block 1, reward learning was significantly negatively correlated with MASQ-positive affect (*r *= −0.28, *p = *.032). When MASQ-positive affect and PTSD-anhedonia was entered into a logistic regression model with learner/non-learner in Block 1 as the dependent variable, MASQ-positive affect was predictive of learning status (OR = 0.93, CI = 0.87–0.99, *p *= .033; see Table 2).

**Table 1.** Descriptive statistics and correlations between measures of anhedonia (MASQ total and subscales) and PTSD-anhedonia cluster (Pietrzak et al., 2015), reward learning and Feedback-Related Negativity (FRN) at electrode Fz.

Measure	*Descriptive statistics: mean* *(SD)*	PTSD-anhedonia	MASQ-loss of interest	MASQ-positive affect*	MASQ-total	Reward learning; Blocks 1–3	Reward learning; Block 1
PTSD-anhedonia	9.80 (3.46)						
MASQ-loss of interest	22.38 (5.26)	0.47 (*p* < .001)					
MASQ-positive affect	51.93 (9.90)	0.56 (*p* < .001)	0.55 (*p* < .001)				
MASQ-total	74.26 (13.65)	0.60 (*p* < .001)	0.79 (*p* < .001)	0.94 (*p* < .001)			
Reward learning 1–3	0.26 (1.55)	0.18 (n.s.)	0.06 (n.s.)	0.14 (n.s.)	0.10 (n.s.)		
Reward learning 1	0.16 (1.04)	−0.02 (n.s.)	−0.14 (n.s.)	−0.28 (*p =* .032)	−0.25 (*p* = .057)	0.32 (*p* = .012)	
Feedback-Related Negativity (FRN), Fz	0.55 (5.80)	0.29 (*p *= .023)	0.02 (n.s.)	0.05 (n.s.)	0.05 (n.s.)	0.09 (n.s.)	0.03 (n.s.)

Reward learning 1–3; response bias developed across all three blocks; Reward learning 1: Response bias developed from first to last half of Block 1 (Pizzagalli et al., 2008). *MASQ-positive affect is reversely scored; higher scores mean less positive affect.

### 3.2. Do PTS-affected veterans who fail to learn from reward produce a shallower FRN compared to veterans who learn from reward?

**Figure 2.**
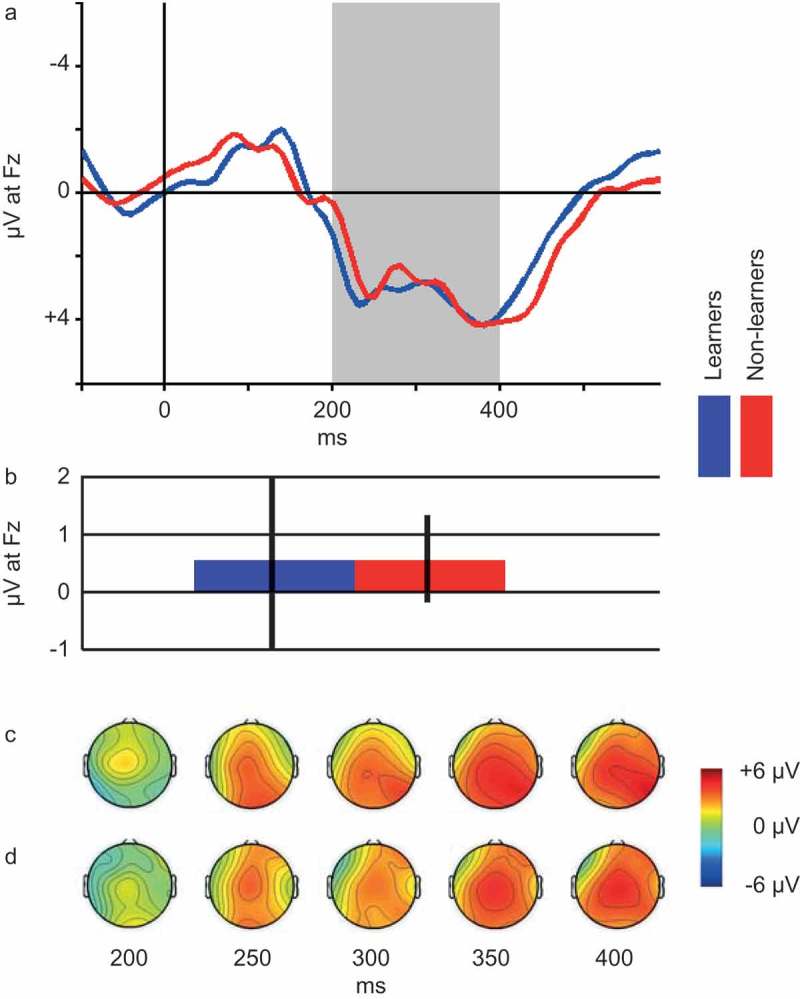
Panel A: Grand average ERP waveforms. Blue line represents reward-learners (*n* = 36), red line represents reward-non-learners (*n* = 25). Shaded area depicts the 200–400 ms interval, based on which the FRN is computed (see Methods section). Panel B: Average FRN for reward Learners (*n* = 36, blue bar) vs. non-learners (*n* = 25, red bar). Note: Error bars represent standard error of the mean. Panel C: Scalp distribution of mean reward ERP generated by learners at select latencies within the FRN interval. Panel D: Scalp distribution of mean reward ERP generated by non-learners at select latencies within the FRN interval.

As described in the Methods section, participants were divided into reward learners and non-learners based on their response bias. Over Blocks 1–3, 45 individuals (73.8%) were categorized as learners based on their performance on the probabilistic reward task. Over the course of Block 1, 36 individuals (59.0%) were categorized as learners, while the reward-non-learners consisted of 25 individuals. Due to a ceiling effect when computing response bias across all three rounds, we proceeded with the categorization of learners and non-learners across Block 1. Grand average ERP-waveforms recorded at site Fz for the two groups can be seen in Figure 2(a) and the scalp distribution of the FRN for reward learners and non-learners can be seen in Figure 2(c,d), respectively. An independent *t*-test comparing FRN between the two groups revealed no significant differences (*t*(59) = 0.006, *p *= .995).

### 3.3. Is level of anhedonia in PTS-affected veterans negatively correlated with FRN?

Correlations between self-reported anhedonia scales and FRN can be seen in Table 2. The MASQ-scales did not correlate with FRN while the PTSD-anhedonia subscale correlated positively with FRN (*r *= 0.29, *p = *.023). When MASQ-positive affect and PTSD-anhedonia were entered into a linear regression model with FRN as the dependent variable, PTSD-anhedonia was significantly related to FRN (β = 0.36, *t*(58) = 2.53, *p *= .022; see Table 2).

**Table 2.** Results of two regression analyses. (A) Logistic regression with learning status in Block 1 (learner/non-learner) as the outcome and (B) Linear Regression with Feedback-Related Negativity (FRN) as the outcome.

	(A) Logistic regression: learner/non-learner		(B) Linear regression: FRN	
	OR (CI)	*p*-value	β	*p*-value
MASQ-PA	0.93 (0.87–0.99)	0.033	−0.15	0.335
PCL-ANH	1.16 (0.96–1.40)	0.134	0.36	0.022
	Nagelkerke R^2^ = 0.11		R^2^**= **0.09	
	Χ^2^(2) = 6.24, *p *= .044		*F*(2,26) = 2.84, *p *= .067	

### 3.4. Exploratory: individual PTSD-anhedonia symptoms and FRN

**Figure 3.**
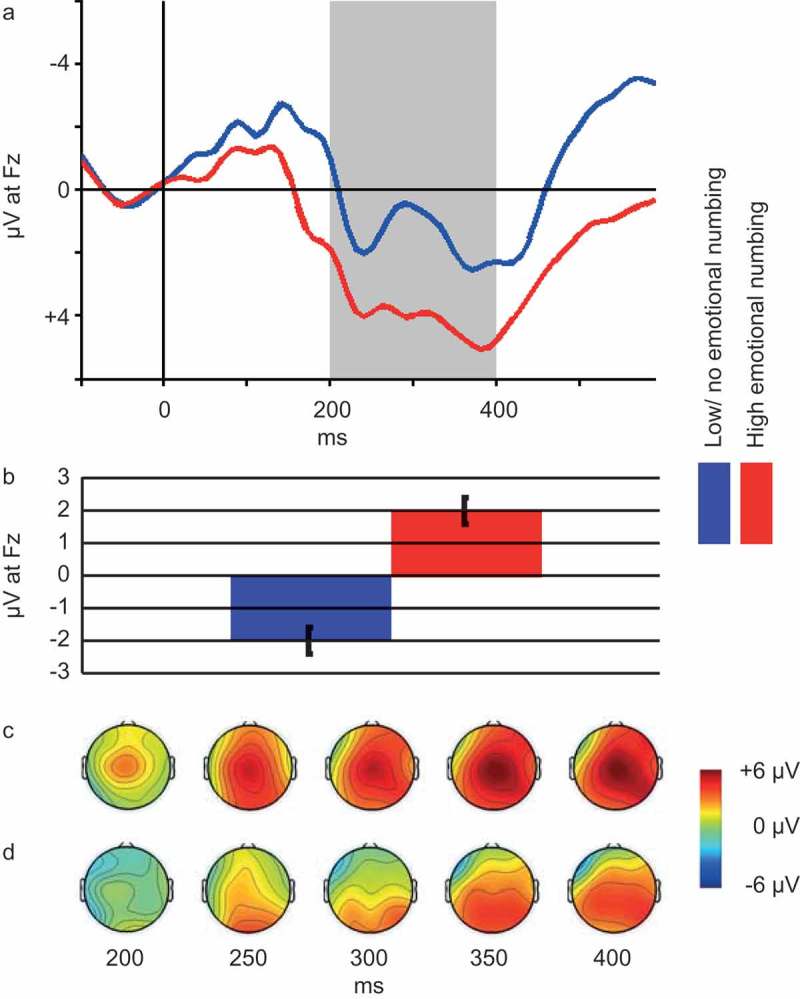
Panel A: Grand average ERP waveforms and scalp plots of the interval 200–400 ms post stimulus. Blue line represents participants with no or low emotional numbing (*n* = 21), red line represents participants with high emotional numbing (*n* = 40). Shaded area depicts the 200–400 ms interval, based on which the FRN is computed (see Methods section). Panel B: Average FRN for participants with high (*n* = 40, red bar) vs. participants with no or low (*n* = 21, blue bar) emotional numbing. Note. Error bars represent standard error of the mean. Panel C: Scalp map of mean reward ERP generated by participants with high emotional numbing (*n* = 40) at select latencies within the FRN interval. Panel D: Scalp map of mean reward ERP generated by participants with no or low emotional numbing at select latencies within the FRN interval.

Based on the correlation between PTSD-anhedonia and FRN, we decided to further explore the relation between individual symptoms in the PTSD-anhedonia symptom cluster and FRN. We found significant correlations between FRN and two out of three symptoms, namely *Detachment or estrangement* (*r *= 0.26, *p *= .046) and *Restricted range of affect* (*r *= 0.38, *p = *.003). Due to the exploratory nature of this analysis, *p*-values were Bonferroni corrected. With this correction, only *Restricted range of affect* remained significantly correlated with FRN. Hence, we proceeded to categorize individuals as having (item score ≥ 3; *n* = 40) or having no or low symptoms of (item score ≤ 2; *n* = 21) restricted range of affect and compared FRN of the two groups (see Figure 3). An independent *t*-test revealed a significant difference in mean FRN between the groups (*t*(60) = 2.72, *p *= .009). Figure 3 represents the ERP at Fz, FRN depth and scalp distribution of relevant portions of the ERP for the two groups.

## 4. Discussion

In this study, we found support for our hypothesis that anhedonia in PTS-affected veterans should correlate negatively with reward learning. We did, however, not find the expected negative correlation between reward learning and FRN, nor between anhedonia as measured by the MASQ and FRN. We found a correlation between PTSD-anhedonia and FRN and, in an exploratory analysis, we found this correlation to be driven exclusively by restricted range of affect. These mixed findings suggest complex relations between symptoms of anhedonia, alterations of reward behaviour and changes in neural responses to reward in a trauma-exposed, treatment-seeking population. This warrants further discussion.

As expected, anhedonia registered by the MASQ-AD instrument showed a moderate correlation with reward-based learning. In agreement with findings of Pizzagalli and colleagues (2008), this relation was only found between the MASQ positive affect subscale and learning within the first block of the experiment. This may indicate a possible performance ceiling inherent in the task design, producing similar learning results after the first block regardless of the presence of anhedonia symptoms. PTSD-anhedonia as measured by the PCL-C instrument, however, did not correlate with behavioural reward-based learning within the complete paradigm nor within the first block. This suggests a difference in the anhedonia constructs registered by the two self-report instruments, and their relation to reward behaviour as measured by the experimental paradigm. A comparison of items contributing to PTSD-anhedonia and constituent items in MASQ positive affect and MASQ loss of interest reveals similarities in registering loss of interest and fatigue or low energy. A key difference separating PTSD-anhedonia from the MASQ positive affect subscale is the inclusion of emotional numbing or restricted range of affect. Thus, PTSD-anhedonia scores may effectively represent a different symptom profile than mirrored in MASQ scores.

When comparing the FRN response to reward, subjects who produced reward-based learning behaviour within the first block did not differ in FRN depth from participants who did not. This is contrary to observations by Santesso and colleagues (2008). Anhedonia symptom-level as registered by the MASQ positive affect scale did not correlate with FRN depth, neither did scores on the MASQ loss of interest scale. Interestingly, level of PTSD-anhedonia correlated weakly with FRN depth.

The inability of the present study to replicate the correlation between FRN and learning behaviour found by Santesso and colleagues (2008) calls for further discussion. Although the relation between neural and behavioural reward responses to reward may seem straightforward, the behavioural and neural parameters compared here may reflect different stages of reward processing. As described in the Introduction, reward processing can be viewed as involving three stages (Berridge & Kringelbach, 2008). First, *reward anticipation*, preceding the rewarding stimulus. Motivation towards achieving reward is involved in *reward learning* or reward-based behaviour maximizing reward outcome. Finally, rewards are enjoyed in a stage of *reward consumption*. Reward-based learning, as produced by the psychophysical task in the current study, reflects a behavioural change due to reward anticipation. In contrast, the FRN response is evoked by receiving reward and therefore linked to reward consumption. Although both are linked to reward, these two stages may reflect separate psychological processes. Thus, in a subject, motivation towards detecting a target efficiently may co-occur with a lack of enjoyment in response to target-related reward, and vice versa. Indeed, previous studies have found that the neural processes of anticipation and consumption of reward can be distinguished using experimental paradigms that carefully accounts for the temporal aspects of reward processing (Novak & Foti, 2015; Rademacher et al., 2010).

These differences in behaviour and neural responses over the time course of reward processing might be relevant to the interpretation of our seemingly conflicting findings: we found no relation between learning and FRN or MASQ-AD and FRN, but we did find a correlation between PTSD-anhedonia and FRN. This motivated an exploratory analysis of the constituent symptoms of PTSD-anhedonia and their relation to the FRN response, revealing a moderate correlation between the item registering emotional numbing and FRN. Given that FRN is evoked as a response to a reward achieved or consumed, a possible interpretation of the current finding is that FRN reflects a consummatory reward response or emotional response upon achieving reward, which correlates negatively with emotional numbing or restricted range of affect.

Anhedonia and emotional numbing are often are clustered together (Pietrzak et al., 2015) but, as suggested by our findings, they may play entirely different roles in responses to traumatic stress. For instance, a reduction in reward responsiveness in the consummatory phase may be accompanied by an increase in reward-anticipating behaviour (Pickett, Bardeen, & Orcutt, 2011). The importance of discriminating between these two stages of reward behaviour and their relation to clinical symptom expressions is highlighted by findings indicating that emotional numbing predicts risk of chronification of PTSD (Malta, Wyka, Giosan, Jayasinghe, & Difede, 2009; Marshall et al., 2006). Current findings suggest a need for further investigations of objective measures of different aspects of reward processing and their relation to traumatic stress.

While abovementioned theoretical and empirical perspectives from the literature offer an explanation for our findings, differences in design between the current study and previous research may also account for the lack of replication of central results from previous studies applying the same paradigm. While the experimental paradigm itself is a close replication of the task employed by Pizzagalli et al. (2005) and Santesso and colleagues (2008), the strategies of participant inclusion were deliberately different. Whereas previous authors investigated differences either between MDD cases and healthy controls or between individuals selected from the extremes of reward-based learning behaviour or lack of said behaviour (i.e. an extreme case design in a healthy population), the current study included all treatment-seeking subjects from a clinic for the duration of study inclusion. Our aim was to test whether differences in anhedonia symptoms could be detected by the reward-based learning paradigm. Thus, participants may have presented with a higher within-group variance of anhedonia symptoms, reward-based learning behaviour, and neural responses to reward. This may account for the lack of group differences.

In many everyday situations, expectancy to the outcome of our efforts, or the pleasure gained from solving a problem or completing a task, may impose meaning into everyday life. As already described, alterations of such reward behaviour may impact key components of psychological functioning, such as the ability to learn or to gain pleasure. Our present data suggest that two distinct component reactions to traumatic stress produces differential changes in this regard. Although emotional numbing and anhedonia may seem similar and be treated collectively as ‘negative alterations of cognition and mood’ (APA, 2013), the psychological functions influenced may be dissimilar. If supported by further research, this may direct attention to differences among trauma patients, as well as towards treatment strategies directed differentially towards these symptoms. Such efforts may potentially increase our abilities to relieve symptoms of traumatic stress.

### 4.1. Limitations

A central finding in the current study is the relation between emotional numbing and FRN. The testing of associations between FRN and individual PTSD symptoms within PTSD anhedonia was done in an exploratory fashion, and as such was not part of the initial hypotheses. We will argue though, that in cross-diagnostic research into clinical phenotypes, a certain degree of exploration is warranted. While the current findings raise important questions, they call for replication in a hypothesis-driven study. Further, emotional numbing was assessed by a single PCL-C item, which is far from optimal. Future studies should include valid measures of both anhedonia and emotional numbing with the purpose of distinguishing the two. Examples of the latter would include the Glover Numbing Questionnaire (Glover, Lader, Walker-O’Keefe, & Goodnick, 1997), the Emotional Reactivity and Numbing Scale (Orsillo, Theodore-Oklota, Luterek, & Plumb, 2007) and a recently developed brief instrument by Frewen and colleagues (Frewen et al., 2012).

A substantial number of initial participants were excluded from the study, due to not fulfilling all rounds of the experiment or because they produced insufficient EEG data. Both might be due to recruiting treatment-seeking veterans at clinical intake. At treatment intake, symptom levels are supposedly peaking, presumably leaving participants fragile to exhaustion and early termination of the session, while also having a potentially negative influence on EEG signal quality. The number of included participants, however, is still sufficient for the performed analyses, and the exclusion rate is within the range of previous ERP research in clinical populations.

Finally, we employed a paradigm where the reward given was monetary. Anhedonia and emotional numbing are often linked to social interactions as they may describe the absence of interest in or feelings towards others. Hence, one could expect that the nature of anhedonia and emotional numbing would be better captured by employment of social reward stimuli such as, for example, faces. Indeed, a recent review of reward functioning in PTSD (Nawijn et al., 2015) suggests that more consistent reward functioning deficiencies are seen in posttraumatic stress related pathology when related to social reward. This calls for further investigation of the distinctive features of anhedonia and emotional numbing in stress-related psychopathology.
